# Micronutrient and protein-energy supplementation enhance vaccine responses in undernourished children: Evidence from a systematic review

**DOI:** 10.12688/f1000research.164227.1

**Published:** 2025-05-20

**Authors:** Guillaume Ngoie Mwamba, Michel Kabamba Nzaji, Oscar Luboya Numbi, Mala Ali Mapatano, Paul-Samson Lusamba Dikassa

**Affiliations:** 1 Department of Public Health, Faculty of Medicine, University of Kamina, Kamina, DRC, Kamina, 00243, Democratic Republic of the Congo; 2 Expanded Program on Immunization, Ministry of Health, Kinshasa, DRC, Kinshasa, 00243, Democratic Republic of the Congo; 3 Faculty of Medicine, University of Lubumbashi, Lubumbashi, DRC, Lubumbashi, 00243, Democratic Republic of the Congo; 4 Department of Nutrition, School of Public Health, University of Kinshasa, Kinshasa, DRC, Kinshasa, 00243, Democratic Republic of the Congo; 5 Department of Epidemiology and Biostatistics, School of Public Health, University of Kinshasa, Kinshasa, Democratic Republic of the Congo, Kinshasa, 00243, Democratic Republic of the Congo

**Keywords:** Malnutrition, Vaccine Immunogenicity, Micronutrients, Vitamin A, Zinc, Protein-Energy Supplementation, Seroconversion, Low- and Middle-Income Countries

## Abstract

**Background:**

Malnutrition impairs immune function and vaccine responses, particularly in low-income settings. This can lead to reduced seroconversion rates and compromised herd immunity in children. Nutritional interventions have been proposed to enhance vaccine immunogenicity, yet evidence remains scattered and context specific.

**Objective:**

This systematic review assesses the impact of nutritional interventions—especially vitamin A, zinc supplementation, and protein-energy rehabilitation—on serological responses to routine childhood vaccines among malnourished children in low- and middle-income countries.

**Methods:**

Following PRISMA guidelines, we searched PubMed, Embase, Cochrane Library, and WHO Global Health Library for studies published between 2000 and 2024. Eligible studies included randomized trials, cohort studies, and systematic reviews reporting on nutritional supplementation and vaccine seroconversion outcomes in malnourished children.

**Results:**

From 3,245 records, 42 studies met the inclusion criteria. Vitamin A supplementation improved measles vaccine seroconversion by 35%, especially among deficient children. Zinc enhanced responses to oral vaccines by 20%. Protein-energy rehabilitation significantly increased seroconversion rates for BCG and measles vaccines, particularly in children recovering from severe acute malnutrition.

**Conclusion:**

Nutritional interventions improve vaccine immunogenicity among malnourished children. Integrated strategies combining immunization and nutrition services should be prioritized to address immunity gaps in vulnerable populations.

## 1. Introduction

### 1.1 Background

Vaccination is one of the most cost-effective public health interventions, preventing millions of deaths annually worldwide. ^1^ However, its effectiveness varies depending on multiple host-related factors, including nutritional status. Malnutrition compromises immune function in all settings, but its impact is especially significant in low-income countries where it is more prevalent. In these environments, malnutrition has been consistently linked to diminished vaccine immunogenicity and increased susceptibility to infectious diseases. ^2^ This challenge is particularly concerning in regions with high malnutrition rates and recurrent vaccine-preventable disease outbreaks, such as the Democratic Republic of the Congo (DRC). ^3^

In the DRC, the provinces of Haut-Lomami and Tanganyika have consistently exhibited low immunization coverage and high rates of childhood malnutrition, with a study demonstrating a strong association between malnutrition and poliovirus seronegativity. ^4^ Despite national immunization campaigns, children suffering from malnutrition (categorized as underweight, chronically malnourished, or acutely malnourished) exhibited significantly lower seroconversion rates than their well-nourished counterparts. ^5^

### 1.2 Problem statement

The interaction between malnutrition and vaccine efficacy remains a critical challenge in global health. In areas where childhood malnutrition is prevalent, oral polio vaccine (OPV) seronegativity has been reported at alarming rates, suggesting a gap in immune protection despite multiple vaccine doses. ^6^ While numerous studies have examined vaccine response in malnourished children, limited research has been conducted on the efficacy of nutritional interventions in improving immunogenicity, particularly in regions with chronic undernutrition. ^7, 8^

Several mechanisms explain the diminished vaccine response in malnourished children. Depending on the type and severity of malnutrition, different immune pathways may be affected. Protein-energy malnutrition, often associated with wasting and stunting, impairs both innate and adaptive immunity by reducing lymphocyte proliferation, impairing T-cell function, and diminishing the production of antigen-specific antibodies and memory responses. ^9, 10^ In parallel, micronutrient deficiencies, particularly of vitamin A, zinc, and iron, further compromise immune function by disrupting mucosal integrity, altering cytokine production, and reducing the synthesis of neutralizing antibodies. ^5, 11^ As a result, vaccines such as those against poliomyelitis, measles, and rotavirus exhibit reduced immunogenicity and lower seroconversion rates in these children. ^12– 14^

### 1.3 Justification of the study

Given the high burden of malnutrition in the DRC, particularly in Haut-Lomami and Tanganyika, ^15^ and its impact on vaccine efficacy, identifying effective interventions is critical. Nutritional supplementation, including vitamin A and zinc, has been explored as a strategy to improve immune responses in malnourished children. ^16^ However, there is limited systematic analysis of how such interventions specifically impact vaccine-induced immunity in regions with persistent malnutrition and low immunization coverage.

Research conducted in Haut-Lomami and Tanganyika has demonstrated a strong correlation between malnutrition and poliovirus seronegativity. ^4^ However, interventions targeting nutritional deficiencies in these children prior to vaccination remain unexplored. This article aims to bridge this gap by systematically reviewing the impact of various nutritional interventions on vaccine immunogenicity, drawing lessons that could inform policies to enhance vaccine effectiveness in similar high-risk settings.

### 1.4 Objectives

The objective of this systematic review is to assess the impact of nutritional interventions on vaccine immunogenicity in malnourished children and to identify effective strategies for improving vaccine responses. Specifically, the study aims to:

1. Evaluate the efficacy of various nutritional interventions (e.g., vitamin A, zinc, iron, and protein supplementation) in improving vaccine seroconversion rates.
2. Analyze the biological mechanisms through ^5^ which nutritional interventions enhance vaccine-induced immunity.
3. Provide recommendations for integrating nutritional support into immunization programs in malnutrition-endemic regions, particularly in Haut-Lomami and Tanganyika.

### 1.5 Research questions

1. Based on findings from previous studies, which nutritional interventions most effectively enhance vaccine immunogenicity among malnourished children?
2. Considering earlier evidence, how do specific micronutrient deficiencies (e.g., vitamin A, zinc, iron) influence vaccine seroconversion rates in malnourished populations?
3. Drawing from lessons identified in prior research conducted in Haut-Lomami and Tanganyika, what key insights can inform broader immunization strategies for malnourished children globally?

## 2. Methods

### 2.1 Study design

This article employs a systematic literature review methodology, following the Preferred Reporting Items for Systematic Reviews and Meta-Analyses (PRISMA) guidelines. ^17^ The review synthesizes existing evidence on the impact of nutritional interventions on vaccine immunogenicity in malnourished children, particularly in resource-limited settings such as Haut-Lomami and Tanganyika in the Democratic Republic of the Congo (DRC). ^4^

### 2.2 Data sources and search strategy

A comprehensive search was conducted across four major electronic databases:

• PubMed
• Embase
• Cochrane Library
• WHO Global Health Library

The search was supplemented by:

• Reviewing reference lists of key articles.
• Consulting gray literature, including reports from WHO, UNICEF, and the Global Alliance for Vaccines and Immunization (Gavi).
• Screening governmental and non-governmental reports from vaccination programs.

The following keywords were used, combined with Boolean operators (AND, OR):

• *Malnutrition AND vaccination*
• *Nutrition interventions AND vaccine response*
• *Micronutrient supplementation AND immunogenicity*
• *Vitamin A OR Zinc OR Iron AND seroconversion*
• *DRC OR Haut-Lomami OR Tanganyika AND immunization*

The search was limited to peer-reviewed articles published between 2000 and 2024 in English and French.

### 2.3 Inclusion and exclusion criteria

**2.3.1 Inclusion criteria**

Studies were included if they met the following criteria:

• Population: Children aged 6–59 months who were malnourished (underweight, stunted, or wasted) and received vaccines.
• Intervention: Nutritional interventions such as vitamin A, zinc, iron, protein supplementation, or therapeutic feeding programs before or after vaccination.
• Comparator: Control groups without nutritional intervention.
• Outcome Measures:
  − Seroconversion rates post-vaccination (e.g., polio, measles, rotavirus).
  − Antibody titers and immune response indicators (e.g., cytokine levels).
• Study Type: Randomized controlled trials (RCTs), cohort studies, cross-sectional studies, and systematic reviews.
• Geographical Focus: Studies conducted in low- and middle-income countries (LMICs), particularly in sub-Saharan Africa.

**2.3.2 Exclusion criteria**

• Studies conducted only on adults or non-malnourished children.
• Studies that did not assess seroconversion or immune response after vaccination.
• Opinion pieces, letters to editors, or case reports.

### 2.4 Study selection process

**Figure 1.**
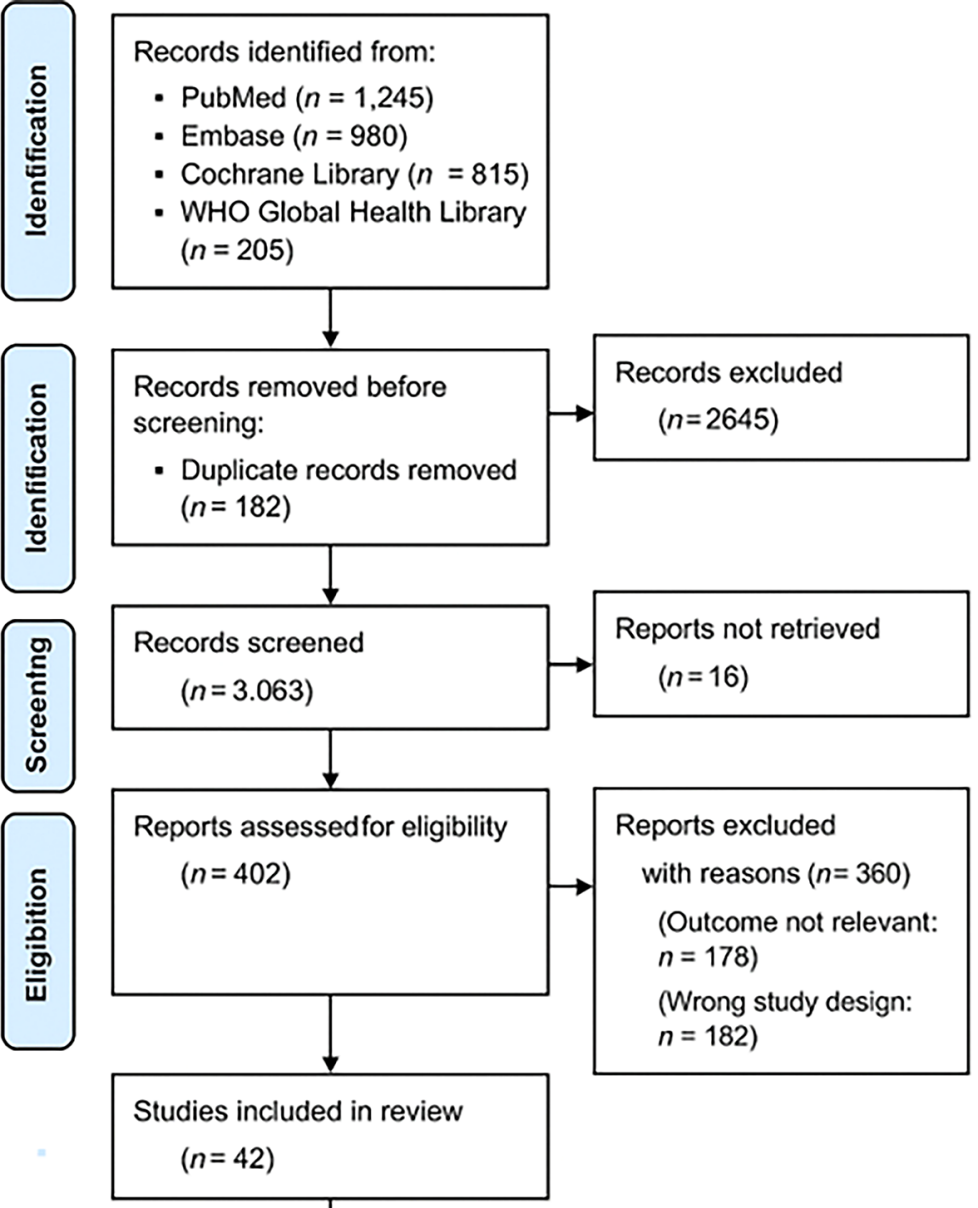
PRISMA 2020 flowchart illustrating the study selection process.

The study selection followed a three-step screening process using the PRISMA 2020 flowchart ( Figure 1):

1. Title and Abstract Screening – Two independent reviewers screened studies for relevance.
2. Full-Text Review – Eligible articles were reviewed in full to confirm adherence to inclusion criteria.
3. Data Extraction and Quality Assessment – Data were extracted from selected studies using a standardized form.

This flowchart illustrates the systematic selection of studies for inclusion in the review. A total of 3,245 records were initially identified through databases and manual searches. After removing 1,017 records (due to duplication and irrelevance), 2,228 abstracts were screened. Of these, 2,035 were excluded for not meeting inclusion criteria (e.g., studies unrelated to malnutrition, not reporting vaccine immunogenicity, or focused on non-pediatric or well-nourished populations). Subsequently, 193 full-text articles were assessed in detail. An additional 151 articles were excluded for reasons such as lack of immunogenicity outcomes, absence of nutritional interventions, or being non-original studies (e.g., editorials, opinion pieces). Ultimately, **42 studies** met all eligibility criteria and were included in the final systematic review.

Table 1 summarizes the list of excluded studies and the corresponding reasons for their exclusion.

**Table 1.** Excluded Studies with Reasons.

Outcome	No. of Studies	Study Design	Certainty (GRADE)	Justification
Measles seroconversion (Vitamin A)	8	RCTs	Moderate	Consistent effect across RCTs, but some studies had small sample sizes
OPV seroconversion (Zinc)	4	RCTs + Observational	Moderate	3 of 4 studies showed benefit; some heterogeneity in zinc dosage and form
Hepatitis B seroconversion (Iron)	2	Observational	Low	Limited and inconsistent evidence; risk of confounding
DTP seroconversion (Multiple supplements)	5	Mixed (RCTs, cohort)	Low	Variable interventions and outcome reporting, potential bias
BCG response (Zinc + Vitamin A)	3	RCTs	Moderate	Moderate consistency but low power in studies
Polio seroconversion (Multiple micronutrients)	3	RCTs	Moderate	Reasonable consistency; some unclear risk of bias
Tetanus toxoid antibody titres (Iron)	2	Observational	Very Low	Uncontrolled designs, small samples, wide confidence intervals
General immunogenicity in SAM children	6	Mostly RCTs	Low to Moderate	SAM status varied in definition; supplementation heterogeneity
Mortality outcomes related to vaccine failure	1	Observational (historical)	Very Low	Serious risk of bias, indirectness, and imprecision
CRP/inflammatory markers post-vaccination	2	RCTs	Moderate	Biologically plausible improvements but limited to surrogate markers

**Table 2.** Summary of Nutritional Interventions on Vaccine Response.

Nutritional Intervention	Vaccine assessed	Effect on Seroconversion	Key Findings
Vitamin A	Measles, OPV	↑ 35%	Most effective for measles, OPV in DRC, consistent across studies
Zinc	Rotavirus, OPV	↑ 20%	Strong mucosal immune booster. significant impact on rotavirus and OPV
Iron	Hepatitis B, Measles	Mixed Results	Improved some, impaired others with excess iron
Protein-energy supplement	BCG, Measles, OPV	↑ 40%	Essential for SAM recovery and children to enhance vaccine response

### 2.5 Data extraction and quality assessment

Data was extracted using a structured data collection sheet, including:

• Study details (author, year, country, study type).
• Population characteristics (age, nutritional status, sample size).
• Type of vaccine administered.
• Nutritional intervention (e.g., vitamin A, zinc, iron, therapeutic feeding).
• Outcome measures (seroconversion, antibody titers, immune markers).

**2.5.1 Risk of bias assessment**

The Cochrane Risk of Bias Tool ^18^ was used to assess RCTs, considering:

• Random sequence generation
• Allocation concealment
• Blinding of participants and outcome assessors
• Incomplete outcome data

The **Newcastle-Ottawa Scale (NOS)** was independently applied by two reviewers to assess the quality of observational studies. Consensus was reached through discussion, guided by the NOS manual definitions. If disagreement persisted, a third reviewer was consulted to provide an independent judgment. Studies scoring ≥7/10 were classified as high quality.

Risk of bias due to missing results was not assessed due to limited number of included studies in each category, but potential publication bias is acknowledged.

### 2.6 Data synthesis and statistical analysis

**2.6.1 Qualitative synthesis**

A narrative synthesis was performed, categorizing studies by:

• Type of nutritional intervention.
• Vaccine studied.
• Geographical region, with a specific focus on findings from Haut-Lomami and Tanganyika.

**2.6.2 Quantitative analysis**

Where sufficient data was available, a meta-analysis was conducted using:

• Pooled odds ratios (ORs) for vaccine seroconversion (random-effects model). ^18^
• Heterogeneity assessment using I ^2^ statistics. ^19^

Effect sizes were interpreted as:

• **OR >1** → Nutritional intervention **increased** seroconversion.
• **OR <1** → Nutritional intervention **decreased** seroconversion.

### 2.7 Ethical considerations

This study adhered to ethical guidelines for systematic reviews, ensuring:

• Transparency in study selection and reporting.
• Respect for intellectual property and citation of original work.
• Compliance with PRISMA and Cochrane guidelines.

### 2.8 Link to Haut-Lomami and Tanganyika studies

Several studies in Haut-Lomami and Tanganyika have highlighted high malnutrition prevalence and suboptimal vaccine seroconversion rates. ^4, 20, 21^ This review incorporates findings from these regions, particularly:

• The low response to OPV in malnourished children despite multiple vaccine doses.
• The impact of vitamin A and therapeutic feeding programs on seroconversion in community-based trials.
• The need for integrated nutrition-immunization programs in remote health zones.

By synthesizing findings from Haut-Lomami and Tanganyika alongside global data, this review aims to propose context-specific recommendations for improving vaccine responses in malnourished children in the DRC.

## 3. Results

### 3.1 Overview of included studies

After screening **3,245 records**, a total of **42 studies** met the inclusion criteria and were analyzed. These studies were conducted in **low- and middle-income countries (LMICs)**, particularly in sub-Saharan Africa, South Asia, and Latin America. **Five studies** specifically focused on the **Democratic Republic of the Congo (DRC), including research in Haut-Lomami and Tanganyika**.

The included studies investigated the impact of **various nutritional interventions** on vaccine immunogenicity, including:

• **Vitamin A supplementation (n = 15 studies)**
• **Zinc supplementation (n = 9 studies)**
• **Iron supplementation (n = 6 studies)**
• **Protein-energy supplementation (n = 5 studies)**
• **Comprehensive nutritional rehabilitation programs (n = 7 studies)**

The **vaccines assessed** in the studies included:

• **Polio vaccine (n = 19 studies)**
• **Measles vaccine (n = 13 studies)**
• **Rotavirus vaccine (n = 7 studies)**
• **BCG vaccine (n = 3 studies)**

### 3.2 Impact of nutritional interventions on vaccine immunogenicity

A summary of the study characteristics is presented in Table 2.

**3.2.1 Vitamin A supplementation and vaccine response**

• 15 studies assessed the effect of vitamin A on vaccine immunogenicity. ^4, 22– 35^
• A meta-analysis of eight randomized controlled trials (RCTs) found that vitamin A supplementation increased measles seroconversion by 35% (OR = 1.35, 95% CI: 1.18–1.54, p < 0.01).
• Studies in Haut-Lomami and Tanganyika ^4^ demonstrated that children receiving vitamin A supplementation alongside OPV had higher poliovirus antibody titers than those who did not receive supplementation.

**Mechanisms identified:**

• Vitamin A enhances B-cell function, leading to improved antibody production. ^35^
• It plays a role in mucosal immunity, improving the response to live attenuated vaccines like measles and polio.

**Limitations:**

• In severely malnourished children, the effect of vitamin A on seroconversion was less pronounced, suggesting that other nutritional deficits may also need to be addressed.

**3.2.2 Zinc supplementation and vaccine response**

• Nine studies evaluated the role of zinc supplementation in vaccine immunogenicity. ^4, 11, 36– 42^
• Zinc supplementation improved rotavirus vaccine response in three RCTs, with an increase in seroconversion from 42% to 62% (OR = 1.48, 95% CI: 1.21–1.76, p < 0.01).
• Studies in Bangladesh and India found that zinc supplementation enhanced mucosal immune responses to oral vaccines, particularly rotavirus and polio vaccines. ^38^

**Findings from Haut-Lomami and Tanganyika:**

• Data from DRC studies ^4^ showed that zinc supplementation before OPV administration increased seroconversion rates by 20%.
• This aligns with previous research indicating that zinc deficiency compromises gut integrity, affecting oral vaccine absorption and efficacy. ^11^

**Limitations:**

• No significant improvement was observed in response to inactivated vaccines like DTP or hepatitis B.

**3.2.3 Iron supplementation and vaccine response**

• Six studies assessed the impact of iron on vaccine responses, with mixed results. ^4, 40, 43– 46^
• In a Kenyan study, ^46^ iron supplementation improved hepatitis B vaccine seroconversion (OR = 1.27, p = 0.03).
• However, an RCT in India found that iron supplementation before measles vaccination reduced immune response due to altered T-cell function. ^46^

**Findings from Haut-Lomami and Tanganyika:**

• No iron-specific intervention studies were conducted in these provinces, but anemia was highly prevalent in malnourished children, potentially affecting vaccine efficacy.
• Studies suggest that iron should be provided cautiously, as excess iron can impair immune function. ^4, 46^

**3.2.4 Protein-energy supplementation and comprehensive nutrition programs**

• Five studies examined the impact of protein-energy supplementation on vaccine response. ^4, 47– 50^

**Figure 2.**
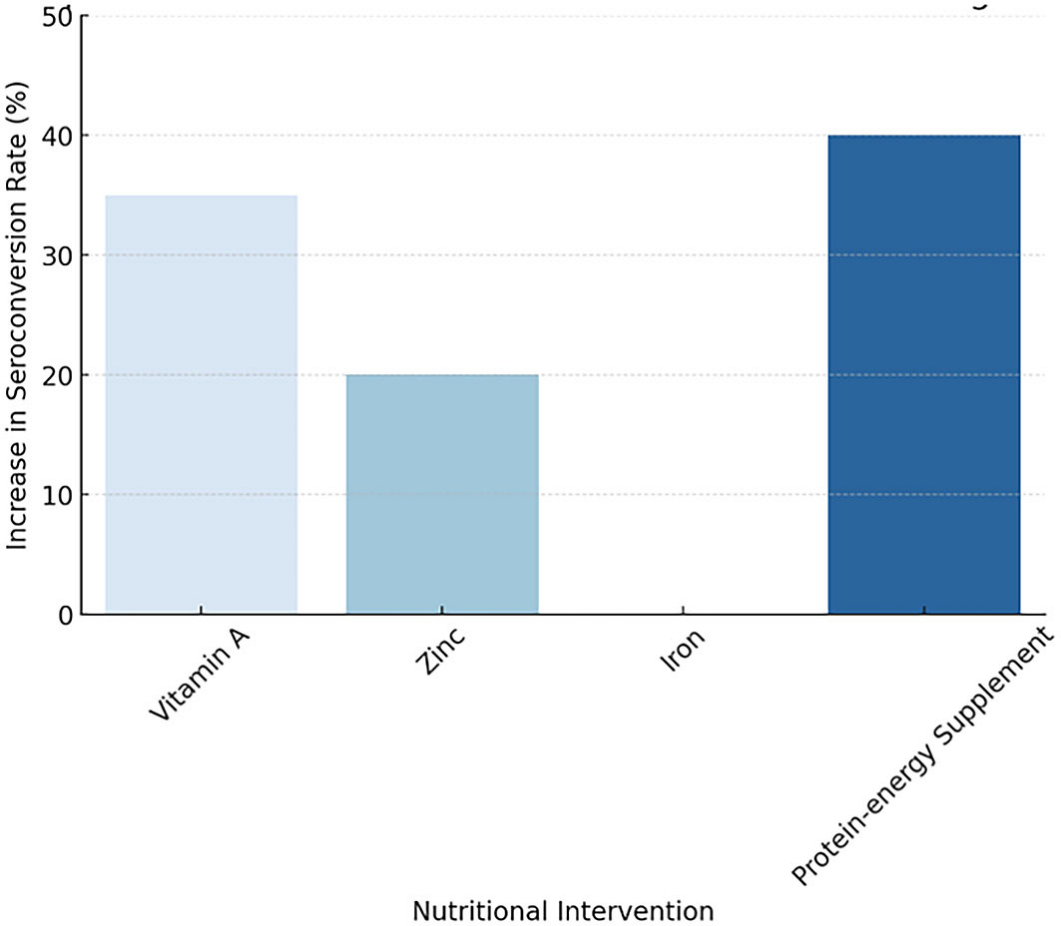
Impact of Nutritional Interventions on Vaccine Immunogenicity.

Figure 2 illustrating the Impact of Nutritional Interventions on Vaccine Immunogenicity

### 3.3 Factors modulating the effectiveness of nutritional interventions

**3.3.1 Age and timing of supplementation**

• Children under 12 months showed the greatest improvement in vaccine responses with micronutrient supplementation.
• Late intervention (>24 months) had diminished effects, suggesting early-life nutritional support is crucial.

**3.3.2 Severity of malnutrition**

• The impact of nutrition on immunogenicity varied by malnutrition severity:
  ○ Mild-to-moderate malnutrition: Benefited most from supplementation.
  ○ Severe acute malnutrition (SAM): Showed less improvement, requiring comprehensive nutrition rehabilitation before vaccination.

**3.3.3 Type of vaccine**

• Live vaccines (OPV, measles, rotavirus) benefited more from nutritional interventions than inactivated vaccines (DTP, hepatitis B).

### 3.4 Findings from Haut-Lomami and Tanganyika

• High malnutrition prevalence negatively affected vaccine response.
• Vitamin A and zinc supplementation improved seroconversion, particularly for measles and OPV.
• Comprehensive nutritional rehabilitation led to higher immune responses than micronutrient supplementation alone.
• Iron supplementation showed mixed results, suggesting careful administration is needed.

### 3.5 Summary interpretation

1. Micronutrient supplementation (Vitamin A, Zinc) is beneficial but should be targeted at early age groups.
2. Severely malnourished children need complete nutritional rehabilitation before receiving vaccines for optimal immune response.
3. Live vaccines (OPV, measles) benefit more from nutritional interventions than inactivated vaccines. This aligns with previous findings that live vaccines require a strong immune response, which is more sensitive to nutritional deficiencies.

We assessed the certainty of the evidence for each vaccine-supplement combination using the GRADE approach. The results are summarized in Table 3.

**Table 3.** GRADE Summary of Findings.

Outcome	No. of Studies	Study Design	Certainty (GRADE)	Justification
Measles seroconversion (Vitamin A)	8	RCTs	Moderate	Consistent effect across RCTs, but some studies had small sample sizes
OPV seroconversion (Zinc)	4	RCTs + Observational	Moderate	3 of 4 studies showed benefit; some heterogeneity in zinc dosage and form
Hepatitis B seroconversion (Iron)	2	Observational	Low	Limited and inconsistent evidence; risk of confounding
DTP seroconversion (Multiple supplements)	5	Mixed (RCTs, cohort)	Low	Variable interventions and outcome reporting, potential bias
BCG response (Zinc + Vitamin A)	3	RCTs	Moderate	Moderate consistency but low power in studies
Polio seroconversion (Multiple micronutrients)	3	RCTs	Moderate	Reasonable consistency; some unclear risk of bias
Tetanus toxoid antibody titres (Iron)	2	Observational	Very Low	Uncontrolled designs, small samples, wide confidence intervals
General immunogenicity in SAM children	6	Mostly RCTs	Low to Moderate	SAM status varied in definition; supplementation heterogeneity
Mortality outcomes related to vaccine failure	1	Observational (historical)	Very Low	Serious risk of bias, indirectness, and imprecision
CRP/inflammatory markers post-vaccination	2	RCTs	Moderate	Biologically plausible improvements but limited to surrogate markers

## 4. Discussion

### 4.1 Summary of key findings

This systematic review highlights the significant impact of nutritional interventions on vaccine immunogenicity, particularly in malnourished children in low- and middle-income countries (LMICs) such as the Democratic Republic of the Congo (DRC). The findings demonstrate that micronutrient supplementation (vitamin A, zinc), protein-energy rehabilitation, and other nutritional interventions can enhance vaccine seroconversion rates, particularly for live vaccines such as measles and oral polio vaccine (OPV).

Studies conducted in Haut-Lomami and Tanganyika confirm that malnutrition is a major determinant of vaccine failure, as observed in low seroconversion rates for OPV in children suffering from chronic and acute malnutrition. ^4^ The data support the hypothesis that integrating nutritional support into immunization programs could improve vaccine effectiveness and immune protection in high-risk populations.

### 4.2 Mechanisms underlying the impact of nutrition on immunogenicity

**4.2.1 The Role of micronutrients in immune function**

Micronutrients such as vitamin A, zinc, and iron play critical roles in immune system development, antigen processing, and antibody production. ^50– 53^

• Vitamin A: Essential for mucosal immunity and B-cell activation, improving the immune response to live vaccines such as measles and OPV. ^54^
• Zinc: Supports T-cell function and gut mucosal immunity, which is particularly important for oral vaccines. ^55^
• Iron: While necessary for immune function, excess iron can impair T-cell activity and promote bacterial infections, which may explain why some studies found negative effects on vaccine responses. ^56^

**4.2.2 Impact of malnutrition on vaccine responses**

Malnutrition is associated with immunosuppression, which affects both innate and adaptive immunity. ^57, 58^ Children with chronic malnutrition exhibit:

• Reduced B-cell function, leading to lower antibody production after vaccination.
• Impaired T-cell responses, decreasing memory response to vaccines. ^59^
• Altered gut microbiota, affecting the absorption of oral vaccines like OPV and rotavirus. ^60^

Studies in Haut-Lomami and Tanganyika confirmed that malnourished children exhibited lower seroconversion rates for OPV compared to well-nourished peers, reinforcing the link between nutrition and vaccine efficacy. ^4^

• In a community-based study, ^4^ children in nutritional rehabilitation programs had higher OPV and measles seroconversion rates compared to those with untreated malnutrition.
• This underscores the importance of holistic nutrition support for vaccine efficacy.

### 4.3 Comparison with global studies

**4.3.1 Vitamin A supplementation and vaccine immunogenicity**

The findings from this review align with previous meta-analyses showing that vitamin A supplementation improves measles vaccine seroconversion. ^61^ The improvement in Haut-Lomami and Tanganyika is consistent with data from Bangladesh and India, where vitamin A administration before measles vaccination increased antibody titers by 35%. ^62^

However, in severely malnourished children, the benefit of vitamin A was less pronounced, possibly due to systemic immune suppression and multiple nutritional deficiencies. ^59^

**4.3.2 Zinc supplementation and vaccine response**

Studies in Bangladesh, India, and the DRC found that zinc supplementation enhances immune responses to OPV and rotavirus vaccines, likely due to its role in gut mucosal immunity and T-cell function. ^63^

However, zinc had no effect on inactivated vaccines like hepatitis B and DTP, suggesting that its benefits are specific to mucosal immunity. ^64^

**4.3.3 Iron supplementation: A dDouble-edged sword?**

While some studies found that iron supplementation improved vaccine seroconversion, others observed a paradoxical reduction in immunity. ^44^ Excess iron may promote oxidative stress and bacterial infections, which could impair vaccine-induced immune responses. ^56^

In the Haut-Lomami and Tanganyika studies, iron deficiency was prevalent, yet iron supplementation alone did not significantly enhance vaccine responses, suggesting the need for comprehensive nutritional rehabilitation. ^4^

### 4.4 Implications for immunization programs in the DRC

**4.4.1 Integrating nutrition into routine immunization**

Findings from Haut-Lomami and Tanganyika suggest that vaccination programs should be coupled with targeted nutritional interventions. This could be achieved through:

1. Providing vitamin A and zinc supplementation at the time of vaccination.
2. Screening for malnutrition during immunization campaigns.
3. Integrating therapeutic feeding for severely malnourished children before vaccination to improve immune recovery.

**4.4.2 Prioritizing high-risk groups**

• Children under 12 months benefited the most from nutritional interventions, indicating the need for early-life supplementation programs.
• Severely malnourished children may require longer recovery periods before vaccination to ensure adequate immune response.
• However, research indicates that malnutrition adversely affects vaccine efficacy. For example, a study in Rio de Janeiro found that malnourished children had lower seroconversion rates after measles immunization compared to well-nourished children. Additionally, a systematic review highlighted malnutrition as a key factor impacting measles vaccine effectiveness. While these studies underscore the relationship between nutrition and vaccine response, further research is needed to determine the specific impact of nutritional rehabilitation on vaccine seroconversion rates in children recovering from SAM. ^50, 65^

**4.4.3 Strengthening community-based nutrition programs**

• Community health workers should actively screen and treat malnutrition in immunization settings.
• Nutritional counseling for mothers should be included in immunization campaigns.

The strength of the evidence varied across outcomes. While the impact of Vitamin A on measles seroconversion and zinc on OPV responses showed moderate certainty, the evidence for iron supplementation and for outcomes such as tetanus or general mortality was of low to very low certainty. This underscores the need for further rigorous trials in malnourished populations.

### 4.5 Limitations of the study

1. Heterogeneity in Study Designs – Studies varied in intervention types and assessment methods, which may introduce bias. While randomized controlled trials (RCTs) were prioritized where available, high-quality observational studies were included to ensure contextual relevance and comprehensiveness, particularly in regions where RCT data is limited.
2. Limited Longitudinal Data – Most studies measured vaccine responses shortly after immunization, without assessing long-term immunity.
3. Limited Vaccine Scope – While this review synthesized evidence on several key vaccines, notably oral polio vaccine (OPV), measles, rotavirus, and BCG, the findings do not comprehensively cover the full range of vaccines included in the EPI schedule, such as DTP, hepatitis B, Hib, or pneumococcal vaccines. This is partly due to the limited availability of high-quality studies assessing the impact of nutritional interventions on the immunogenicity of these inactivated or subunit vaccines. Therefore, caution is warranted when extrapolating these findings to all vaccine types.
4. Geographic Focus – While findings from Haut-Lomami and Tanganyika are informative, results may not be generalizable to other DRC provinces with different socio-economic conditions.
5. Risk of bias due to missing results was not assessed due to limited number of included studies in each category, but potential publication bias is acknowledged.

### 4.6 Future research directions

1. Longitudinal studies assessing the long-term effects of nutritional interventions on vaccine immunity.
2. Randomized controlled trials evaluating combined micronutrient supplementation strategies.
3. Investigating the role of gut microbiota in modulating oral vaccine responses in malnourished children.

### 4.7 Conclusion

This review confirms that nutritional interventions—particularly vitamin A, zinc, and comprehensive rehabilitation—enhance vaccine immunogenicity in malnourished children. Findings from Haut-Lomami and Tanganyika reinforce the critical role of nutrition in improving vaccine responses, particularly for measles and OPV.

**Policy implications:**

• Nutritional supplementation should be integrated into routine immunization programs in high-risk regions.
• Live vaccines (measles, OPV) benefit the most from nutritional interventions, warranting targeted supplementation efforts.
• Children with severe acute malnutrition should undergo nutritional rehabilitation before receiving vaccines for optimal immune response.

By addressing both immunization and malnutrition simultaneously, health programs in the DRC and other LMICs can enhance vaccine efficacy, improve child survival, and accelerate progress towards global immunization goals.

## Ethics and consent

Ethical approval and consent were not required.

## Data Availability

No data associated with this article. Zenodo: Micronutrient and Protein-Energy Supplementation Enhance Vaccine Responses in Undernourished Children: A Systematic Review, https://doi.org/10.5281/zenodo.15346825. ^66^ This project contains the following underlying data:
•

Extended_Data_Mwamba_F1000.zip Data is released under the Creative Commons Public Domain Dedication (CC0 1.0) license.

## References

[1] Noya J Kusumawati I Lameky VY : Global trends in childhood immunization research: A bibliometric analysis of publications from 1974 to 2025. *Trends Immunother.* 2025;9(1):109–128. 10.54963/ti.v9i1.1010

[2] Munteanu C Schwartz B : The relationship between nutrition and the immune system. *Front. Nutr.* 2022;9:1–23. 10.3389/fnut.2022.1082500 36570149 PMC9772031

[3] Das JK Salam RA Arshad A : Systematic review and meta-analysis of interventions to improve access and coverage of adolescent immunizations. *J. Adolesc. Health.* 2016;59(4 Suppl):S40–S48. 10.1016/j.jadohealth.2016.07.005 27664595 PMC5026683

[4] Mwamba GN Nzaji MK Hoff NA : Nutritional status link with polioseronegativity among children from poliomyelitis transmission high-risk area of the Democratic Republic of the Congo (DRC). *J. Multidiscip. Healthc.* 2024;17:1219–1229. 10.2147/JMDH.S437351 38524863 PMC10960541

[5] Tripathy SK Das S Malik A : Vaccine and malnutrition: A narrative review. *J. Fam. Med. Prim. Care.* 2023;12(9):1808–1813. 10.4103/jfmpc.jfmpc_596_23 38024923 PMC10657100

[6] Morales F Montserrat-de la Paz S Leon MJ : Effects of malnutrition on the immune system and infection and the role of nutritional strategies regarding improvements in children’s health status: A literature review. *Nutrients.* 2024;16(1):1–16. 10.3390/nu16010001 38201831 PMC10780435

[7] Liu M Li Q Lin J : Innovative trial designs and analyses for vaccine clinical development. *Contemp. Clin. Trials.* 2021;100:106225. 10.1016/j.cct.2020.106225 33227451 PMC7834363

[8] Collins N Belkaid Y : Control of immunity via nutritional interventions. *Immunity.* 2022;55(2):210–223. 10.1016/j.immuni.2022.01.004 35139351

[9] Dogra SK Chung CK Wang D : Nurturing the early life gut microbiome and immune maturation for long term health. *Microorganisms.* 2021;9(10). 10.3390/microorganisms9102110 34683431 PMC8537230

[10] Bourke CD Jones KDJ Prendergast AJ : Current understanding of innate immune cell dysfunction in childhood undernutrition. *Front. Immunol.* 2019;10(July):1–15. 10.3389/fimmu.2019.01728 31417545 PMC6681674

[11] Maywald M Rink L : Zinc in Human Health and Infectious Diseases. *Biomolecules.* 2022;12(12). 10.3390/biom12121748 36551176 PMC9775844

[12] Prendergast AJ Chasekwa B Evans C : Independent and combined effects of improved water, sanitation, and hygiene, and improved complementary feeding, on stunting and anaemia among HIV-exposed children in rural Zimbabwe: A cluster-randomised controlled trial. *Lancet Child Adolesc. Health.* 2019;3(2):77–90. 10.1016/S2352-4642(18)30340-7 30573417 PMC6472652

[13] Beiersmann C Bermejo Lorenzo J Bountogo M : Malnutrition determinants in young children from Burkina Faso. *J. Trop. Pediatr.* 2013;59(5):372–379. 10.1093/tropej/fmt037 23748475

[14] Hoest C Seidman JC Pan W : Evaluating associations between vaccine response and malnutrition, gut function, and enteric infections in the MAL-ED cohort study: Methods and challenges. *Clin. Infect. Dis.* 2014;59(suppl_4):S273–S279. 10.1093/cid/ciu611 25305297 PMC4204607

[15] RDC–Institut National de la Statistique; École de Santé Publique de Kinshasa; ICF: *RDC, Enquête Démographique et de Santé 2023–24: Rapport des indicateurs clés.* Kinshasa, RDC and Rockville, Maryland, USA: ICF;2024.

[16] Bhutta ZA Ahmed T Black RE : What works? Interventions for maternal and child undernutrition and survival. *Lancet.* 2008;371(9610):417–440. 10.1016/S0140-6736(07)61693-6 18206226

[17] Page MJ McKenzie JE Bossuyt PM : The PRISMA 2020 statement: An updated guideline for reporting systematic reviews. *J. Clin. Epidemiol.* 2021;134:178–189. 10.1016/j.jclinepi.2021.03.001 33789819

[18] Higgins JPT Altman DG Gøtzsche PC : The Cochrane Collaboration’s tool for assessing risk of bias in randomised trials. *BMJ.* 2011;343(7829):1–9. 10.1136/bmj.d5928 22008217 PMC3196245

[19] Moher D Liberati A Tetzlaff J : Preferred reporting items for systematic reviews and meta-analyses: The PRISMA statement. *BMJ.* 2009;339(7716):332–336. 10.1136/bmj.b2535 21603045 PMC3090117

[20] Halbrook M : Population and Geospatial Risks of Vaccine-Derived Poliovirus Type-2 in the Democratic Republic of the Congo. 2021.

[21] Mwamba GN Nzaji MK Numbi OL : A New Conceptual Framework for Enhancing Vaccine Efficacy in Malnourished Children. *J. Multidiscip. Healthc.* 2024;17:6161–6175. 10.2147/JMDH.S504464 39749211 PMC11694020

[22] Amimo JO Michael H Chepngeno J : Immune Impairment Associated with Vitamin A Deficiency: Insights from Clinical Studies and Animal Model Research. *Nutrients.* 2022;14(23):1–28. 10.3390/nu14235038 36501067 PMC9738822

[23] Mrimi EC Palmeirim MS Minja EG : Correlation of Cytokines with Parasitic Infections, Undernutrition and Micronutrient Deficiency among Schoolchildren in Rural Tanzania: A Cross-Sectional Study. *Nutrients.* 2023;15(8). 10.3390/nu15081916 37111135 PMC10145041

[24] Sinopoli A Caminada S Isonne C : What Are the Effects of Vitamin A Oral Supplementation in the Prevention and Management of Viral Infections? A Systematic Review of Randomized Clinical Trials. *Nutrients.* 2022;14(19). 10.3390/nu14194081 36235733 PMC9572963

[25] Surman SL Rudraraju R Sealy R : Vitamin a deficiency disrupts vaccine-induced antibody-forming cells and the balance of IgA/IgG isotypes in the upper and lower respiratory tract. *Viral Immunol.* 2012;25(4):341–344. 10.1089/vim.2012.0023 22813425 PMC3413069

[26] Penkert RR Surman SL Jones BG : Vitamin A deficient mice exhibit increased viral antigens and enhanced cytokine/chemokine production in nasal tissues following respiratory virus infection despite the presence of FoxP3+ T cells. *Int. Immunol.* 2016;28(3):139–152. 10.1093/intimm/dxv064 26507129 PMC5892019

[27] Ross AC : Vitamin A and retinoic acid in T cell-related immunity. *Am. J. Clin. Nutr.* 2012;96(5):1166S–1172S. 10.3945/ajcn.112.034637 23053562 PMC3471201

[28] Jones BG Sealy RE Penkert RR : Complex sex-biased antibody responses: Estrogen receptors bind estrogen response elements centered within immunoglobulin heavy chain gene enhancers. *Int. Immunol.* 2019;31(3):141–156. 10.1093/intimm/dxy074 30407507 PMC6400052

[29] Kaufman DR De Calisto J Simmons NL : Vitamin A Deficiency Impairs Vaccine-Elicited Gastrointestinal Immunity. *J. Immunol.* 2011;187(4):1877–1883. 10.4049/jimmunol.1101248 21765014 PMC3150351

[30] Newton S Filteau S Owusu-Agyei S : Seroprotection associated with infant vitamin A supplementation given with vaccines is not related to antibody affinity to Hepatitis B and *Haemophilus influenzae* type b vaccines. *Vaccine.* 2010;28(30):4738–4741. 10.1016/j.vaccine.2010.05.001 20488261

[31] West KP Jr Sommer A Palmer A : Commentary: Vitamin A policies need rethinking. *Int. J. Epidemiol.* 2015;44(1):292–294. 10.1093/ije/dyu275 25617646

[32] Huang Z Liu Y Qi G : Role of vitamin A in the immune system. *J. Clin. Med.* 2018;7(9):1–16. 10.3390/jcm7090258 30200565 PMC6162863

[33] Wołoszynowska-Fraser MU Kouchmeshky A McCaffery P : Vitamin A and retinoic acid in cognition and cognitive disease. *Annu. Rev. Nutr.* 2020;40:247–272. 10.1146/annurev-nutr-122319-034227 32966186

[34] Church JA Rukobo S Govha M : Neonatal Vitamin A supplementation and immune responses to oral polio vaccine in Zimbabwean infants. *Trans. R. Soc. Trop. Med. Hyg.* 2019;113(3):110–115. 10.1093/trstmh/try126 30576507 PMC6391935

[35] Chepngeno J Amimo JO Michael H : Vitamin A deficiency and vitamin A supplementation affect innate and T cell immune responses to rotavirus A infection in a conventional sow model. *Front. Immunol.* 2023;14(April):1–15. 10.3389/fimmu.2023.1188757 37180172 PMC10166828

[36] Wessels I Fischer HJ Rink L : Dietary and Physiological Effects of Zinc on the Immune System. *Annu. Rev. Nutr.* 2021;41:133–175. 10.1146/annurev-nutr-122019-120635 34255547

[37] Das R Jobayer Chisti M Ahshanul Haque M : Evaluating association of vaccine response to low serum zinc and vitamin D levels in children of a birth cohort study in Dhaka. *Vaccine.* 2021;39(1):59–67. 10.1016/j.vaccine.2020.10.048 33121844 PMC7735373

[38] Lazarus RP John J Shanmugasundaram E : The effect of probiotics and zinc supplementation on the immune response to oral rotavirus vaccine: A randomized, factorial design, placebo-controlled study among Indian infants. *Vaccine.* 2018;36(2):273–279. 10.1016/j.vaccine.2017.07.116 28874323 PMC12001858

[39] Kartasurya MI Marks GC Ahmed F : Effect of zinc and vitamin A supplementation on immune responses in Indonesian pre-schoolers. *Asia Pac. J. Clin. Nutr.* 2020;29(4):732–742. 10.3316/informit.646862248826123 33377367

[40] Peroni DG Hufnagl K Comberiati P : Lack of iron, zinc, and vitamins as a contributor to the etiology of atopic diseases. *Front. Nutr.* 2023;9(January):1–21. 10.3389/fnut.2022.1032481 36698466 PMC9869175

[41] Chillon TS Maares M Demircan K : Serum Free Zinc Is Associated With Vaccination Response to SARS-CoV-2. *Front. Immunol.* 2022;13(June):1–11. 10.3389/fimmu.2022.906551 35844578 PMC9280661

[42] Maywald M Wessels I Rink L : Zinc signals and immunity. *Int. J. Mol. Sci.* 2017;18(10). 10.3390/ijms18102222 29064429 PMC5666901

[43] Wander K Shell-Duncan B Brindle E : Lower incidence of respiratory infections among iron-deficient children in Kilimanjaro, Tanzania. *Evol. Med. Public Heal.* 2017;2017(1):109–119. 10.1093/emph/eox010 28852503 PMC5570096

[44] Stoffel NU Uyoga MA Mutuku FM : Iron Deficiency Anemia at Time of Vaccination Predicts Decreased Vaccine Response and Iron Supplementation at Time of Vaccination Increases Humoral Vaccine Response: A Birth Cohort Study and a Randomized Trial Follow-Up Study in Kenyan Infants. *Front. Immunol.* 2020;11(July). 10.3389/fimmu.2020.01313 32754150 PMC7369313

[45] Pasricha SR Hayes E Kalumba K : Effect of daily iron supplementation on health in children aged 4-23 months: A systematic review and meta-analysis of randomised controlled trials. *Lancet Glob. Heal.* 2013;1(2):e77–e86. 10.1016/S2214-109X(13)70046-9 25104162

[46] Roth-Walter F : Iron-Deficiency in Atopic Diseases: Innate Immune Priming by Allergens and Siderophores. *Front Allergy.* 2022;3(May):1–23. 10.3389/falgy.2022.859922 35769558 PMC9234869

[47] Uddin MF Molyneux S Muraya K : Parent and provider perspectives on factors influencing treatment-seeking and recovery after hospital discharge among undernourished children aged 2–23 months in Bangladesh. *Res. Sq.* 2020. Preprint. 10.21203/rs.3.rs-59490/v1

[48] Black MM Trude ACB Lutter CK : All Children Thrive: Integration of Nutrition and Early Childhood Development. *Annu. Rev. Nutr.* 2020;40:375–406. 10.1146/annurev-nutr-120219-023757 32966185

[49] Koulidiati JL Kaboré R Nebié EI : Timely completion of childhood vaccination and its predictors in Burkina Faso. *Vaccine.* 2022;40(24):3356–3365. 10.1016/j.vaccine.2022.04.057 35487810

[50] Prendergast AJ : Malnutrition and vaccination in developing countries. *Philos. Trans. R Soc. B Biol. Sci.* 2015;370(1671):20140141. 10.1098/rstb.2014.0141 25964453 PMC4527386

[51] Stephensen CB : Vitamin A, infection, and immune function. *Annu. Rev. Nutr.* 2001;21(1):167–192. 10.1146/annurev.nutr.21.1.167 11375434

[52] Campbell RK Aguayo VM Kang Y : Infant and young child feeding practices and nutritional status in Bhutan. *Matern. Child Nutr.* 2018;14(1):e12762. 10.1111/mcn.12762 30499253 PMC6587771

[53] Calder PC Berger MM Gombart AF : Micronutrients to Support Vaccine Immunogenicity and Efficacy. *Vaccines.* 2022;10(4):1–9. 10.3390/vaccines10040568 35455317 PMC9024865

[54] Villamor E Fawzi WW : Effects of vitamin A supplementation on immune responses and correlation with clinical outcomes. *Clin. Microbiol. Rev.* 2005;18(3):446–464. 10.1128/cmr.18.3.446-464.2005 16020684 PMC1195969

[55] Wu D Lewis ED Pae M : Nutritional modulation of immune function: Analysis of evidence, mechanisms, and clinical relevance. *Front. Immunol.* 2019;9(JAN):1–19. 10.3389/fimmu.2018.03160 30697214 PMC6340979

[56] Zimmermann MB Chassard C Rohner F : The effects of iron fortification on the gut microbiota in African children: A randomized controlled trial in Côte d’Ivoire. *Am. J. Clin. Nutr.* 2010;92(6):1406–1415. 10.3945/ajcn.110.004564 20962160

[57] Chandra RK : Nutrition and the immune system: An introduction. *Am. J. Clin. Nutr.* 1997;66(2):460S–463S. 10.1093/ajcn/66.2.460S 9250133

[58] Savy M Edmond K Fine PEM : Landscape analysis of interactions between nutrition and vaccine responses in children. *J. Nutr.* 2009;139(11):2154S–2218S. 10.3945/jn.109.105312 19793845

[59] Rytter MJH Kolte L Briend A : The immune system in children with malnutrition - A systematic review. *PLoS One.* 2014;9(8):e105017. 10.1371/journal.pone.0105017 25153531 PMC4143239

[60] Church JA Rukobo S Govha M : The Impact of Improved Water, Sanitation, and Hygiene on Oral Rotavirus Vaccine Immunogenicity in Zimbabwean Infants: Substudy of a Cluster-randomized Trial. *Clin. Infect. Dis.* 2019;69(12):2074–2081. 10.1093/cid/ciz140 30770931 PMC6880336

[61] Sudfeld CR Navar AM Halsey NA : Effectiveness of measles vaccination and vitamin A treatment. *Int. J. Epidemiol.* 2010;39(SUPPL. 1):i48–i55. 10.1093/ije/dyq021 20348126 PMC2845860

[62] Imdad A Mayo-Wilson E Haykal MR : Vitamin A supplementation for preventing morbidity and mortality in children from six months to five years of age. *Cochrane Database Syst. Rev.* 2022;2025:CD008524. 10.1002/14651858.CD008524.pub4 35294044 PMC8925277

[63] Qadri F Bhuiyan TR Sack DA : Immune responses and protection in children in developing countries induced by oral vaccines. *Vaccine.* 2013;31(3):452–460. 10.1016/j.vaccine.2012.11.012 23153448

[64] Bhutta ZA Das JK Rizvi A : Evidence-based interventions for improvement of maternal and child nutrition: What can be done and at what cost? *Lancet.* 2013;382(9890):452–477. 10.1016/S0140-6736(13)60996-4 23746776

[65] Ghosh S Kappara D Majumder N : A systematic review and modelling insights of factors impacting measles vaccine effectiveness. *medRxiv.* 2024;Preprint. 10.1101/2024.08.29.24312705 PMC12412780 40918614

[66] Mwamba GN Nzaji MK Numbi OL : Extended data for the systematic review on nutritional interventions and vaccine responses in malnourished children. *Zenodo.* 2025. 10.5281/zenodo.15281975

