# Supplementary material for: Micronutrient and protein-energy supplementation enhance vaccine responses in undernourished children: Evidence from a systematic review
Source: F1000Res. 2025 Sep 11;14:507. Originally published 2025 May 20. [Version 4] doi: 10.12688/f1000research.164227.4 (PMC12334918; doi:10.12688/f1000research.164227.4)
Supplement: Peer review history

# Reviewer report: Reviewer response for version 3

**DOI:** 10.5256/f1000research.185041.r399143

**Published:** 2025-08-08

**Authors:** Philip C Calder

**Affiliations:** 1 University of Southampton, Southampton, England, UK

This is the third version of this article. I reviewed the previous version but not the original version. Most of the comments I raised previously have been attended to. However Section  3.2 still begins with the statement "A summary of the study characteristics is presented in Table 2." This is not the case: Table 2 reports the findings, not the characteristics. They are reported in Table S1.

Are the rationale for, and objectives of, the Systematic Review clearly stated?

Yes

Is the statistical analysis and its interpretation appropriate?

Yes

If this is a Living Systematic Review, is the ‘living’ method appropriate and is the search schedule clearly defined and justified? (‘Living Systematic Review’ or a variation of this term should be included in the title.)

Not applicable

Are sufficient details of the methods and analysis provided to allow replication by others?

Yes

Are the conclusions drawn adequately supported by the results presented in the review?

Yes

Reviewer Expertise:

Nutritional immunology

I confirm that I have read this submission and believe that I have an appropriate level of expertise to confirm that it is of an acceptable scientific standard.

# Reviewer report: Reviewer response for version 3

**DOI:** 10.5256/f1000research.185041.r399142

**Published:** 2025-08-08

**Authors:** Eknath D. Ahire

**Affiliations:** 1 MET's Institute of Pharmacy, Nashik, India

It appears that the author has made all the necessary changes in this updated manuscript. Therefore, I have no issues and recommend proceeding with the article to the next step.

Are the rationale for, and objectives of, the Systematic Review clearly stated?

Yes

Is the statistical analysis and its interpretation appropriate?

Yes

If this is a Living Systematic Review, is the ‘living’ method appropriate and is the search schedule clearly defined and justified? (‘Living Systematic Review’ or a variation of this term should be included in the title.)

No

Are sufficient details of the methods and analysis provided to allow replication by others?

Yes

Are the conclusions drawn adequately supported by the results presented in the review?

Yes

Reviewer Expertise:

Nutraceuticals, Pharmaceuticals

I confirm that I have read this submission and believe that I have an appropriate level of expertise to confirm that it is of an acceptable scientific standard.

# Reviewer report: Reviewer response for version 3

**DOI:** 10.5256/f1000research.185041.r401076

**Published:** 2025-08-08

**Authors:** Maren Johanne Heilskov Rytter

**Affiliations:** 1 Slagelse Hospital, Slagelse, Denmark

Review of "Micronutrient and protein-energy supplementation enhance vaccine responses in undernourished children: Evidence from a systematic review [version 3; peer review: 2 approved with reservations]"

 This is an interesting subject of high clinical relevance, addressing the well-known but poorly understood vicious cycle between malnutrition and infection. Specifically the review aims to assess wether supplementation with micronutrients or protein-energy supplements may improve vaccine responses.

 However, the manuscript in its present form does not reliably address the subject in a scientifically sound way: The main problem is that only few of the 42 studies included in the review are clinical studies and provide specific data to answer the study questions. Most of the included articles are reviews, of which the majority are not systematic, but narrative, and as such they do not even fall within the inclusion criteria stated for this review: 

 This is highly problematic, because it makes it unclear what the real origin of the results cited are. For example among the nine studies that the authors state assess the effect of zinc only one is an interventional study (an RCT of probiotics and zinc, which did not find any effect of zinc supplementation on antibody responses) while the remaining are observational studies assessing the effect of zinc on general immune response (but not vaccine response (n=1), assess the association between measured plasma level of zinc and vaccine response (n=2), assess the association of vaccine response to generel nutritional status but not reporting anything about zinc (n=1) or are narrative reviews not reporting any original data (n=3). Accordingly it is nor clear what the origin of the effect estimate given is - and also there is no reference given for this

 The same goes for the remaining sections of the manuscript: it is very unclear what the origin of the conclusions are: for example, in the section about "protein-energy supplementation and comprehensive nutrition programmes" two of the five studies cited are reviews, and the other three are not assessing the effect of nutritional supplementation on antibody response. This may be due to a citation error, and in that case it should be corrected.

 To improve the manuscript I suggest to use a more selective inclusion strategy and NOT include reviews but only studies reporting original data to answer your research question. You may use reviews during your search proces to identify any original studies that are cited in the reviews, which you may have missed during your initial search, but included these only and not the review themselves, as you risk reporting somebody's interpretation of the data (or maybe their hypotheses, or opinions) rather than real data.

 Also, I suggest that you for each topic preset the relevant included studies in one or two tables, outlining the study year, country of origin, number and kind of included children, the study design, the intervention and comparator, the outcome(s) measured, and (perhaps in a separate table) describe the main findings, effect size, and any concern for bias. This will make your findings more transparent for readers.

 Also you should include a discussion of the difficulties in interpreting observational studies regarding malnutrition and immune response, due to the fact that most children with malnutrition also have infections, and elevated acute phase response, which is known to reduce response to vaccines by themselves. Also, most measured  plasma markers of nutrients (including zinc, vitamin D, , ferritin etc) are also acute phase reactants, meaning that any association seen between these markers and immune responses may result from confounding from ongoing infection. This means that all observational studies should be interpreted with caution, and treatment or policy decisions should be based on results from randomised trials (which are less likely to be confounded)

 I also suggest that you, as a safety measure, do not include any studies in your review by Chandra RK, who has published extensively on the relationship between immune function and nutrition, but who has been found to fabricate data and make research fraud. Some of his work has been retracted, but not all of it. Se Smith : Investigating the previous studies of a fraudulent author. Smith R 2005 [Ref 1]

 Finally, I strongly disagree with your statement in the conclusion: "Children with severe acute malnutrition should undergo nutritional rehabilitation before receiving vaccines for optimal immune response."

 First, while malnourished children may have subtle decreases in vaccine responses, there is no data indicating that vaccination does not protect them from disease, and no data suggesting that they are at higher risk of vaccine-related adverse events (provided that they do not have advanced HIV or primary immunodeficiency).

 Second, children with malnutrition are at much higher risk of dying of they get infections, indicating that they are the most likely to benefit from protection from vaccination. For this reason, measles vaccinations have been an important part of nutritional rehabilitation programmes in many places (also because a measles outbreak in a nutritional rehabilitation centre is likely to be disastrous). Se further arguments in the excellent review (also cited by you!) by Prendergast AJ, 2015 [Ref 1]

 I would be happy to review a revised version of your manuscript, but I am convinced that it needs a major revision to be suitable for indexing.

Are the rationale for, and objectives of, the Systematic Review clearly stated?

Partly

Is the statistical analysis and its interpretation appropriate?

I cannot comment. A qualified statistician is required.

If this is a Living Systematic Review, is the ‘living’ method appropriate and is the search schedule clearly defined and justified? (‘Living Systematic Review’ or a variation of this term should be included in the title.)

Not applicable

Are sufficient details of the methods and analysis provided to allow replication by others?

No

Are the conclusions drawn adequately supported by the results presented in the review?

No

Reviewer Expertise:

I am a paediatrician, mainly doing clinical research. In my PhD I worked on the relationship between malnutrition infections and immune function, by assessing the effect of nutritional rehabilitation on thymus size in children with severe and moderate acute malnutrition. I also did a systematic review of the relationship between malnutrition and immune parameters.

I confirm that I have read this submission and believe that I have an appropriate level of expertise to state that I do not consider it to be of an acceptable scientific standard, for reasons outlined above.

## References

[1] : Investigating the previous studies of a fraudulent author. *BMJ* .2005;331(7511) : 10.1136/bmj.331.7511.288 288-291 10.1136/bmj.331.7511.288 PMC118127416052023

[2] : Malnutrition and vaccination in developing countries. *Philosophical Transactions of the Royal Society B: Biological Sciences* .2015;370(1671) : 10.1098/rstb.2014.0141 10.1098/rstb.2014.0141 PMC452738625964453

# Response

**Published:** 2025-09-07

**Authors:** Guillaume NGOIE MWAMBA

**Affiliations:** Ministry of Health, Programme Elargi de Vaccination, Kinshasa, Democratic Republic of the Congo

**Notes to Reviewers – Response to Dr. Maren Johanne Heilskov Rytter**

 Manuscript title: Micronutrient and protein-energy supplementation enhances vaccine responses in undernourished children: Evidence from a systematic review

 Version: 3 → 4

 Reviewer: Dr. Maren Johanne Heilskov Rytter, Slagelse Hospital, Denmark

 General Note

 We sincerely thank the reviewer for the constructive and clinically insightful feedback. We have substantially revised the manuscript to address all concerns. Below is our detailed point-by-point response.

**Inclusion of narrative reviews.**

 Reviewer comment: Most of the included articles are narrative reviews and do not fit the eligibility criteria.

 Response: All narrative reviews were removed. The revised dataset now contains only randomized controlled trials, cohort, and observational studies (29 in total). The PRISMA flow diagram, Abstract, Methods, and Results have been updated accordingly.

**Scientific rigor in the evidence base**

 Reviewer comment: The manuscript does not reliably address the subject in a scientifically sound way.

 Response: The Results were rebuilt to focus exclusively on primary evidence. Table 1 (Excluded studies), Table 2 (Key findings), and Table 3 (GRADE summary) were added to transparently present study-level data and certainty of evidence.

**Confounding in observational studies**

 Reviewer comment: Associations may be confounded by acute infection; biomarkers are acute-phase reactants.

 Response: The Discussion and Limitations were revised to highlight confounding by infection and the acute-phase behavior of biomarkers such as ferritin, zinc, and vitamin D.

**Fraudulent studies (Chandra RK)**

 Reviewer comment: Studies by Chandra RK must be excluded.

 Response: All studies by Chandra RK were excluded. This is clearly stated in the Methods and shown in the PRISMA diagram under 'other reasons'.

**Conclusion on sequencing vaccination**

 Reviewer comment: The statement suggesting nutritional rehabilitation before vaccination is potentially harmful.

 Response: The Conclusion was rewritten to emphasize that vaccination should never be delayed in malnourished children. Immunization and nutrition support should be delivered concurrently.

**Methods detail (PRISMA 2020 alignment)**

 Reviewer comment: The methodology is insufficiently detailed for replication.

 Response: The Methods were expanded to include eligibility criteria, search strategy, dual screening, risk-of-bias assessment, and reasons for excluding reviews. A PRISMA 2020 flow diagram is now included.

**Statistical analysis and meta-analysis**

 Reviewer comment: Statistical validation is required; a meta-analysis should be attempted, or reasons given for not conducting one.

 Response: A statistician reviewed the dataset. Owing to high heterogeneity across interventions, outcomes, and study designs, a global meta-analysis was not feasible. However, a targeted meta-analysis of vitamin A and measles seroconversion (8 RCTs) was performed. Other outcomes were synthesized narratively using the SWiM framework.

**Publication years of included trials**

 Reviewer comment: Several included studies are old.

 Response: Although our initial cutoff was 2000, we retained select pre-2000 trials (e.g., on protein-energy supplementation) that provided unique primary data. Their inclusion is justified in the Methods, and they were critically appraised for risk of bias and interpreted with caution.

# Reviewer report: Reviewer response for version 2

**DOI:** 10.5256/f1000research.183761.r393821

**Published:** 2025-06-28

**Authors:** Philip C Calder

**Affiliations:** 1 University of Southampton, Southampton, England, UK

This manuscript reports a systematic review and partial meta-analysis of trials examining supplementation of vitamin A, zinc, iron and protein/energy on vaccine responses in children. This is a really important topic given global prevalence of protein/energy and micronutrient deficiencies in children and the existence of a high pathogen burden. This seems to be a revision of a previous version but i did not review that. I found this to be very well written article. It is found the vitamin A and zinc and protein/energy repletion increase response to several vaccines; findings with iron were inconsistent. The discussion includes health policy implications of the work.

 Specific comments:

 1. Why was the search limited to 2000 onwards. Please justify.

 2.The PRISMA chart (Figure 1) and text do not seem to match.

 3. Table 2 does not show study characteristics as stated; it seems to summarise the findings.

 4. Section 2.3.3 says meta-analysis was not possible yet there is a meta-analysis of effects of vitamin A.

Are the rationale for, and objectives of, the Systematic Review clearly stated?

Yes

Is the statistical analysis and its interpretation appropriate?

Yes

If this is a Living Systematic Review, is the ‘living’ method appropriate and is the search schedule clearly defined and justified? (‘Living Systematic Review’ or a variation of this term should be included in the title.)

Not applicable

Are sufficient details of the methods and analysis provided to allow replication by others?

Yes

Are the conclusions drawn adequately supported by the results presented in the review?

Yes

Reviewer Expertise:

Nutritional immunology

I confirm that I have read this submission and believe that I have an appropriate level of expertise to confirm that it is of an acceptable scientific standard, however I have significant reservations, as outlined above.

# Response

**Published:** 2025-07-13

**Authors:** Guillaume NGOIE MWAMBA

**Affiliations:** Ministry of Health, Programme Elargi de Vaccination, Kinshasa, Democratic Republic of the Congo

**Response to Reviewer Comments**

**Manuscript Title:** *Micronutrient and protein-energy supplementation enhance vaccine responses in undernourished children: Evidence from a systematic review*

**Manuscript ID:** f1000research-14-507

**Reviewer:** Dr. Philip C. Calder

**Date of Review:** June 28, 2025

 We sincerely thank Dr. Calder for his thoughtful review, kind words, and constructive feedback. We have carefully considered all comments raised and implemented the relevant revisions in the manuscript. Below are our detailed, point-by-point responses.

**Reviewer Comment 1**

*"Why was the search limited to 2000 onwards. Please justify."*

**Response:**

 Thank you for this observation. We have now provided a clear justification in **Section 2.2 **of the revised manuscript. The search was limited to studies published from 2000 onwards due to the substantial evolution in global immunization policy and child nutrition strategies over the past two decades. This includes the launch of the *Global Immunization Vision and Strategy (GIVS, 2006–2015)*, expanded vaccine introduction in low- and middle-income countries (LMICs), and major WHO/UNICEF nutrition initiatives. These developments influenced both research context and methodological consistency, ensuring that data included in this review are more relevant, comparable, and aligned with current global standards.

**Reviewer Comment 2**

*"The PRISMA chart (Figure 1) and text do not seem to match."*

**Response:**

 We appreciate this important remark. We have carefully revised Figure 1 (PRISMA) and ensured full alignment with the narrative description in **Section 2.4** of the manuscript. Discrepancies in the number of records screened and excluded at various stages were corrected. The final version now accurately reflects the number of studies included (n=42) and the corresponding exclusion rationale, bringing the figure and text into consistency.

**Reviewer Comment 3**

*"Table 2 does not show study characteristics as stated; it seems to summarise the findings."*

**Response:**

 Thank you for this helpful clarification. Table 2 has now been re-labeled as **“Key Findings from Included Studies”** to better reflect its content. In response to the need for detailed study characteristics, we have created a separate file titled **Supplementary Table S1 – Study Characteristics**, which includes:

  

- Study ID
- Country/Region
- Nutritional Intervention
- Vaccine Assessed
- Author and Year
- Study Design
- Sample Size
- Age Range
- Outcome
- Reference (with DOI or PMID)

This file is hosted on **Zenodo** as part of our living evidence synthesis initiative and will be periodically updated. The DOI for this supplementary dataset is: https://doi.org/10.5281/zenodo.15873743

**Suggested Manuscript Insertions:**

- *Methods or Results section:* “Detailed study characteristics are provided in Supplementary Table S1 (Zenodo DOI: 10.5281/zenodo.15873743).”
- *Data Availability section:* “Supplementary Table S1 is available on Zenodo and includes complete metadata for each study. The dataset will be updated as new studies become available.”

**Reviewer Comment 4**

*"Section 2.3.3 says meta-analysis was not possible yet there is a meta-analysis of effects of vitamin A."*

**Response:**

 We acknowledge this oversight and have revised Section 2.3.3 to clarify that a comprehensive meta-analysis across all interventions was not feasible due to clinical and methodological heterogeneity. However, we were able to perform a **targeted meta-analysis** for a subset of studies examining the effect of **vitamin A supplementation on measles vaccine seroconversion**, where study designs and outcome metrics were sufficiently comparable. This partial analysis is now clearly described, and limitations (e.g., absence of forest plots and publication bias assessments) are transparently noted in the Results and Limitations sections.

 We are grateful to Dr. Calder for his insightful feedback, which has greatly enhanced the quality and clarity of our systematic review. We trust that the revisions satisfactorily address all concerns.

**Sincerely,**

 Dr. Guillaume Ngoie Mwamba (on behalf of all co-authors)

 ORCID: https://orcid.org/0000-0003-0460-877X

# Reviewer report: Reviewer response for version 1

**DOI:** 10.5256/f1000research.180700.r388286

**Published:** 2025-06-13

**Authors:** Eknath D. Ahire

**Affiliations:** 1 MET's Institute of Pharmacy, Nashik, India

**General Comments**

 This is a timely and well-structured systematic review addressing a critical issue in global public health—how nutritional interventions can improve vaccine responses in malnourished children. The authors have employed standard PRISMA guidelines, incorporated a range of high-quality studies, and made relevant policy recommendations. The manuscript is clearly written, logically organized, and makes a valuable contribution to the field.

 Specific comments: 

 1. Please include forest plots and funnel plots to visually display the results of the meta-analysis and assess heterogeneity and publication bias.

 2. Clarify the process used for GRADE rating assignment—was this done independently by reviewers or through consensus? This will improve transparency.

 3. Expand the discussion of risk of bias, particularly regarding publication bias, selective outcome reporting, and missing data within included studies.

 4. Tables 2 and 3 could benefit from clearer visual presentation. Consider bolding statistically significant findings or using shading/highlighting to improve readability.

 5. Briefly describe the contents and purpose of the extended dataset available via Zenodo in the methods section to help readers understand its role.

 6. Tighten the writing in the background and discussion sections to reduce repetition and improve clarity.

 7. Ensure consistent use of terms such as "seroconversion," "vaccine immunogenicity," and "antibody response" throughout the manuscript.

 8. Check for consistency in reference formatting according to Vancouver style; a few entries need adjustment.

 9. Indicate whether the systematic review was registered (e.g., on PROSPERO). If not, briefly explain why this step was omitted.

 10. More clearly state that the findings predominantly apply to live vaccines (e.g., OPV, measles) due to limited data on inactivated vaccines.

 11. Although DRC is a focus, consider expanding policy recommendations to other LMICs with similar malnutrition and immunization challenges.

 12. Discuss in more detail how the timing of nutritional interventions (pre-, concurrent with, or post-vaccination) influences vaccine responses.

Are the rationale for, and objectives of, the Systematic Review clearly stated?

Yes

Is the statistical analysis and its interpretation appropriate?

Yes

If this is a Living Systematic Review, is the ‘living’ method appropriate and is the search schedule clearly defined and justified? (‘Living Systematic Review’ or a variation of this term should be included in the title.)

No

Are sufficient details of the methods and analysis provided to allow replication by others?

Yes

Are the conclusions drawn adequately supported by the results presented in the review?

Yes

Reviewer Expertise:

Nutraceuticals, Pharmaceuticals

I confirm that I have read this submission and believe that I have an appropriate level of expertise to confirm that it is of an acceptable scientific standard, however I have significant reservations, as outlined above.

# Response

**Published:** 2025-06-16

**Authors:** Guillaume NGOIE MWAMBA

**Affiliations:** Ministry of Health, Programme Elargi de Vaccination, Kinshasa, Democratic Republic of the Congo

Point-by-Point Response to Reviewer 1 Comments

 Manuscript Title: Micronutrient and protein-energy supplementation enhance vaccine responses in undernourished children: A systematic review

 Manuscript ID: 164227

 Corresponding Author: Dr. Guillaume Ngoie Mwamba

 Dear Editor,

 We are grateful for the constructive feedback provided by Reviewer 1. Below is our point-by-point response detailing how each suggestion was addressed in the revised manuscript.

 Reviewer Comment

 Author Response

 Action Taken

 1.Include forest and funnel plots to visually display results.

 Thank you. A meta-analysis was not conducted due to heterogeneity in study designs, intervention types, outcomes, and effect measures. We clarified this in the manuscript.

 Clarified in Section 2.3 (Data Synthesis).

 2.Clarify the process used for GRADE rating assignment.

 GRADE ratings were independently assigned by two reviewers; discrepancies were resolved through consensus.

 Added in Section 2.4 (Quality Assessment).

 3.Expand the discussion of risk of bias.

 We have expanded the discussion to address publication bias, selective outcome reporting, and missing data across included studies.

 Expanded in Discussion, Paragraph 5.

 4.Improve visual presentation of Tables 2 and 3.

 Tables 2 and 3 have been revised for clarity. Statistically significant findings are now bolded.

 Tables revised for visual clarity.

 5.Briefly describe the Zenodo dataset in the Methods section.

 We added a brief description of the extended dataset (PRISMA checklist, flowchart, summary table, CRediT matrix) in the Methods.

 Added in Section 2.5 (Extended Data).

 6.Tighten the writing in background and discussion sections.

 Both sections were streamlined to reduce redundancy and improve clarity.

 Revised Introduction and Discussion sections.

 7.Ensure consistent terminology throughout the manuscript.

 We revised the manuscript to ensure consistent use of terms such as “seroconversion,” “vaccine immunogenicity,” and “antibody response.”

 Terminology harmonized across the manuscript.

 8.Check reference formatting for Vancouver style.

 All references were reviewed and updated to follow Vancouver formatting guidelines.

 References updated accordingly.

 9.Indicate whether the review was registered in PROSPERO.

 The review was registered in PROSPERO (ID: CRD420251058388). This is now reported in the Methods section and cited in the References.

 New subsection added: “Protocol Registration” in Methods and reference #18 added.

 10.Clarify that findings predominantly apply to live vaccines.

 We now explicitly state that the findings mainly concern live vaccines (e.g., OPV, measles), due to limited evidence on inactivated vaccines.

 Added in Results and expanded in Discussion.

 11.Expand policy implications to other LMICs.

 We broadened the policy recommendations in the Conclusion to apply to other LMICs beyond the DRC.

 Revisions made in the Conclusion section.

 12.Discuss the timing of nutritional interventions.

 We added discussion on how timing (pre-, co-, or post-vaccination) of supplementation influences vaccine response.

 Added in Discussion, Paragraph 6.

 We sincerely thank the reviewer for their thoughtful insights, which helped us improve the manuscript significantly. We trust the revised version meets the journal's expectations and remain at your disposal for any further revisions.

 Kind regards,

 Dr. Guillaume Ngoie Mwamba

 On behalf of all co-authors

 Email: guillaumengoiemwamba@gmail.com

 ORCID: https://orcid.org/0000-0003-0460-877X
